# Long-acting antipsychotic treatments: focus on women with schizophrenia

**DOI:** 10.1177/20451253241263715

**Published:** 2024-07-31

**Authors:** Sofia Brissos, Vicent Balanzá-Martínez

**Affiliations:** Centro Hospitalar Psiquiátrico de Lisboa, Av. Brasil 53, Lisbon 1700, Portugal; Teaching Unit of Psychiatry and Psychological Medicine, Department of Medicine, CIBERSAM, INCLIVA, University of Valencia, Valencia, Spain

**Keywords:** breastfeeding, contraception, long-acting injectable antipsychotics, menopause, postpartum, pregnancy, schizophrenia, sex

## Abstract

Effective management of schizophrenia (SZ) requires long-term treatment with antipsychotics (APs) to prevent clinical relapse, attain remission and improve patients’ personal and social functioning, and quality of life. Although APs remain the cornerstone treatment for patients with SZ, despite their potential benefits, long-acting injectable APs (LAI-APs) remain underused, most notably in women with SZ. The efficacy and tolerability of APs differ significantly between men and women, and some of these differences are more noticeable depending on the patient’s age and the stage of the disorder. Although sex differences may influence treatment outcomes in SZ, their pertinence has been insufficiently addressed, especially regarding the use of LAI-APs. Some biological and social experiences, such as pregnancy, lactation, contraception and menopause, are specific to women, but these remain under-researched issues. Implications of this disorder in parenting are also of special pertinence regarding women; therefore, taking sex differences into account when treating SZ patients is now recommended, and improving personalized approaches has been proposed as a priority in the management of psychosis. In this narrative, critical review, we address some aspects specific to sex and their implications for the clinical management of women with SZ, with a special focus on the potential role of LAI-AP treatments.

## Plain language summary (OPTION 1)

• Schizophrenia is a chronic mental illness, and patients often need to take antipsychotic medications in the long-run in order to stay well, avoid re-occurrence of symptoms and improve their everyday functioning and quality of life.

• Antipsychotics are available in both pill and injection form. The latter is known as long-acting injectable antipsychotics (LAI-APs) and can be administered from weekly to twice a year.

• Despite their effectiveness and practicality due to less frequent administration, LAI-APs remain largely underused, especially in women with schizophrenia.

• The efficacy and tolerability of antipsychotics can be very different between men and women, and some of these differences may be more pronounced depending on the patient’s age and the phase of the illness.

• Notably, physical and social aspects such as pregnancy, lactation, contraception, parenting and menopause and their effects on the treatment with antipsychotics and particularly LAI-APs in women with schizophrenia are under-studied.

• Nevertheless, we have now become more aware of the importance of these sex differences, and it is recommended to take them routinely into consideration when treating patients with schizophrenia in clinical practice.

• In this article, we discuss how factors specific to sex can influence the treatment of women with schizophrenia and focus on the potential role of LAI-AP medications.

## Introduction

Effective management of schizophrenia (SZ) requires early intervention and continuous long-term (maintenance) treatment to reduce symptoms, maintain functionality, improve quality of life^1^ and prevent relapse.^2^ Antipsychotics (APs) remain the cornerstone treatment for this disorder, both in the short-, and in the long-term; however, soon after their development in the 1950s, poor adherence to oral formulations was found to be a critical issue, and this led to the development in 1966 of the first long-acting injectable AP (LAI-AP) fluphenazine enanthate.^3^ Since then, several first- and second-generation LAI-APs have become available, and although clinical trials in the 1970s showed their use resulted in a dramatic reduction in SZ morbidity,^3,4^ over the years LAI-APs have been over clouded by concerns of more side effects, lack of efficacy, feelings of coercion, and being seen as an attempt by psychiatrists to impose a treatment without due regard for patients’ feelings or rights.^3,5^

Treatment guidelines have been very conservative on the use of LAI-APs,^6,7^ especially regarding their early implementation,^6^ even though some already state that ‘patients should be offered the option of depot/long-acting injectable AP medication for maintenance treatment, given the evidence of lower risk of relapse’.^8^

Despite their benefits, LAI-APs remain underutilized in patients with SZ, most notably in women, both in the United States and Europe.^9–12^ This may be because women with SZ usually show better help-seeking behaviour than men,^13^ and are thought of as more adherent to treatment,^14^ because women may experience some side effects as more severe than men, and due to preferences of both the physician and the patient. In addition, it may be due to the disproportionate inclusion of men in clinical trials^15^ and a lack of sex-based analyses.^16^ Indeed, in a recent review covering trials with the LAI-APs formulations of risperidone, paliperidone and aripiprazole women constituted only 36% of the total number of patients, and a separate analysis of the primary study outcome between men and women was carried out in only 6 of the 40 works, with only 3 of 40 trials discussing results separately by sex.^16^ Moreover, and despite international recommendations, none of the studies followed a hormonal interaction approach or analyzed the potential interaction of LAI-APs with other drugs such as hormonal contraceptives.^16^ The dearth of information regarding the use of LAI-APs in (a potential) pregnancy might also explain clinician concerns.

Men and women are different, and sex differences in SZ are nowadays scientifically established in the clinic^14^ and epidemiological surveys.^17^ These differences are usually attributed to female hormones, which also act as neuromodulators.^18^ The oestrogen protection hypothesis posits that the action of oestrogens in the brain affects mood, cognition and behaviour, which in turn may explain women’s apparent advantages over men with regard to SZ prognosis.^19^ Moreover, the efficacy and the tolerability of APs are significantly different between men and women with SZ,^12^ and some of these differences become more noticeable depending on the age of the person and the stage of the disease.^20^ On the other hand, some of these differences may (also) be attributable to cultural factors^21,22^ that change with time, place and circumstance.

Although sex differences may influence the success of SZ treatment, their pertinence has been insufficiently addressed,^14^ especially regarding LAI-AP use.^16^ Targeting sex differences, improving personalized approaches and prolonging the observation period at early intervention services have been proposed as a priority in the management of psychosis,^23^ since there are biological and social experiences, such as pregnancy, lactation and menopause, that are specific to women (Summary Table 1).

**Summary Table 1.** General considerations.

General considerations
• Despite their proven benefits, LAI-APs remain underused in individuals with SZ, especially in women. • Sex differences may influence treatment outcomes in SZ, but this is not usually considered when prescribing LAI-APs. • Specificities regarding pregnancy, lactation, contraception, parenting and menopause remain under-researched. • It is recommended that the treatment of SZ considers sex differences. This represents a priority in the management of this illness and aligns with the growing emphasis on the personalization of treatments. • Future clinical trials should compare the efficacy of LAI-APs between men and women with SZ, as well as between pre- and postmenopausal women, stratifying outcomes by sex and hormonal status, respectively. • Analysis of side effects by sex will help clarify risks and improve safe prescribing of LAI-APs in clinical practice.

LAI-APs, long-acting injectable antipsychotics; SZ, schizophrenia.

In this nonsystematic, narrative, critical review, we address some aspects specific to sex and their implications for the clinical management of women with SZ, with a special focus on the (potential) role of LAI-AP treatments.

## Methods

The present article was planned as a narrative overview of the literature, comprising a critical discussion of available evidence. We aimed to provide a reference for wider reading instead of a systematic review^24^). Thus, no systematic literature search was attempted.

Electronic databases (PubMed/MEDLINE, Science Direct and Google Scholar) were searched up to 20 October 2023. Search terms related to gender (e.g. ‘gender’, ‘sex’), the disorder and its treatment (e.g. ‘schizophrenia’, ‘psychosis’, ‘antipsychotics’, ‘long acting’, ‘depot’) and women’s issues (e.g. ‘menopause’, ‘lactation’, ‘breastfeeding’, ‘contraception’, ‘postpartum’, ‘parenting’) were used. The electronic search was supplemented by hand-searching of reference lists of reviews to locate further suitable publications. We included human observational and experimental studies on the relationship between psychosis/APs and gender/sex issues. We considered only published articles and excluded the grey literature and articles not published in English.

## Findings

### Sex differences with APs

#### Sex differences in the pharmacokinetics and pharmacodynamics of APs

Body composition differs significantly between men and women, the latter having smaller organs, more fatty tissue and less muscle tissue, which in turn changes the volume of distribution, especially for lipophilic drugs, such as APs.^14^ However, because of the action of female hormones on fat cells, this difference decreases after menopause.^25^ Women have 10%–15% greater blood flow to the brain, which makes it easier for a drug to reach their target receptor.^26^ Therefore, women generally have 20%–30% higher dose-adjusted concentrations, which is a proxy for bioavailability, compared to men.^27^

Moreover, women’s stomachs are less acidic than men’s, and this increases the absorption of APs in oral formulation.^28^ APs’ absorption and distribution is also influenced by P-glycoprotein, which is some two-fold lower in women due to oestrogen and progesterone,^29^ enabling the drug to enter the brain more easily and potentially causing more side effects.^30^ Concerning excretion, kidney function is lower in women, and this might be particularly relevant for APs such as amisulpride, sulpiride and paliperidone, leading to higher plasma levels in women.

Regarding LAI-APs, a higher amount of subcutaneous fat slows the absorption and perfusion of these formulations,^25^ leading to more body accumulation. Therefore, relapse may occur later after discontinuation of LAI-APs in women, even though side effects may take longer to dissipate. On the other hand, lipophilic LAI-AP, such as paliperidone, may not need to be administered to women as frequently as to men,^18,31^ spacing administrations and possibly simplifying even more the therapeutic regimen.

Regarding APs’ pharmacodynamics, oestrogens influence the levels of dopamine transporters and receptors in cortical and striatal regions of the brain, resulting in higher D2 receptor occupancy in women compared to that in men, even when plasma concentrations are similar; however, this changes in postmenopausal women, who need higher dosages to reach the same efficacy.^12^

Despite these differences, very little information is available regarding pharmacodynamic differences between men and women treated with LAI-APs, and this may have negative implications in clinical practice.

### Sex differences in treatment response and tolerability to APs

A better response to most APs has been reported in women, but that depends on their age. Premenopausal women do respond better, but women after 45, and especially in the age range of 65–69 show a worse clinical course and may need more hospitalizations.^32^

Regarding side effects, women show an increased prevalence of QT interval prolongation induced by many APs, leading to increased incidence of tachycardias, arrhythmias and syncope in women.^33^ Women also show an increased prevalence of extrapyramidal side effects, especially with postmenopausal oestrogen decline.^12^ Tardive dyskinesia is also more common in women, although the incidence is relatively low in premenopausal women (3%–5%), possibly due to the protective antioxidant effects of oestrogens.^34^

APs often cause hyperprolactinaemia,^35^ which can result in galactorrhoea, cessation of normal cyclic ovarian function and hirsutism.^36^ Premenopausal women have physiologically higher levels of prolactin and are closer to the threshold for hyperprolactinaemia.^37^ Prolactin secretion induces oestrogen deficiency, which can lead to polycystic ovarian syndrome, infertility, osteoporosis, sexual dysfunction and an increased risk of breast cancer.^36^ Prolactin-sparing APs (e.g. aripiprazole, cariprazine) may, therefore, be preferred over prolactin-raising ones (e.g. amisulpride, sulpiride, haloperidol, risperidone, paliperidone) in the treatment of women with SZ.

Weight gain and abdominal adiposity are also more frequent in women taking APs^38^; therefore, it is important that research examining AP-induced metabolic side effects analyses outcomes by sex to identify the mechanisms and help clarify the risks, to improve safe prescribing of these medications.^39^ Special attention should be paid to clozapine and olanzapine, and APs that induce minimal weight gain may be preferred.^12^

A final note to osteoporosis: patients with SZ have a high risk of low bone mineral density (BMD) which is doubled after age 50, with osteoporosis being reported to affect 9% of men and 21% of women.^14^ It has been assumed that AP-induced hyperprolactinaemia and the reduction of oestrogens and androgens might be the link between SZ and low BMD seen in some patients.^14^ Since gonadal hormones increase BMD, the link to SZ has been assumed to be AP-induced hyperprolactinaemia leading to reduction of oestrogens and androgens.^14^ The situation may be, however, more complicated, since a genetic link may exist between the two diseases,^40^ and factors such as high rates of smoking, obesity, stress, lack of exercise, poor nutrition and alcohol consumption, also play a role.^14^ Therefore, there is some controversy about whether the use of prolactin-sparing APs prevents the drop of BMD in individuals with SZ.^41^

#### Sex differences in the prescription of APs

Although there are no sex-specific clinical guidelines for prescribing APs, there are differences in prescription patterns between men and women, which seem to be related with preferences of both the physician and the patient.^12^ Women may experience some side effects as more severe than men, for example, AP-induced weight gain,^42^ which in turn may influence the decision to take medication, causing nonadherence to prescribed medications in women.^43,44^ In that sense, priority should be given to APs with less potential for weight gain, especially in women.

Although therapeutic adherence is usually thought of as higher in women,^42^ in fact it seems to be similar to men.^22,27,45^ In fact, in a recent large study, female sex was a risk factor for treatment discontinuation among patients on LAI risperidone and paliperidone.^46^ Low treatment adherence is a major risk factor for relapses in SZ, and LAI formulations are posited as an effective treatment option to minimize that risk. Despite this, in the United States and Europe, the prescription rates of LAI-APs are lower in women.^9–11,47^

#### Sex differences regarding patients’ preferences of AP treatment

Clinicians may view LAIs for only the most chronically and severely ill patients, or for problematic/doubtful adherent patients who are not truthful about their treatment adherence.^48^ Combined with anxiety over medication side effects or the use of needles, this can make patients apprehensive about LAI-APs, requiring that clinicians adopt a thoughtful approach to present LAI-APs as a treatment option.^49^

Patients with experience taking LAI-APs have different perceptions of the injectable formulations than patients taking oral APs. Indeed, that group experiences less anxiety and depression, and feel more energetic,^50^ with the majority stating that side effects are milder and that relapse prevention/efficacy is better.^51^

We are not aware of studies specifically addressing attitudes between men and women with SZ towards LAI-APs, and whether there are significant sex differences. A Chinese study found that sex plays no significant difference in the willingness to accept LAI-APs,^52^ whereas in a Greek study only female gender was significantly associated with LAI-AP use.^53^ However, as these authors posited, this does not necessarily mean that women have a more positive attitude towards LAI-APs or that they are more frequently eligible for such treatment, since women could just be more easily forced by caregivers to accept such treatment.^53^

Regarding LAI-AP administration, women have been shown to be more likely than men to prefer administration by a clinician of the same gender (34% vs 12%),^54^ but there seems to be no gender differences regarding preference for site of administration, with both men (60%) and women (57%) preferring deltoid injections.^55^ Thus, availability of LAI-APs as deltoid and gluteal injections is perceived as an important advantage by patients and psychiatrists alike and is expected to enhance LAI acceptability,^56^ namely by women. Nevertheless, since women have lower muscle mass and more lipid deposits at injection sites, different needle lengths should be considered, especially to avoid site sores and/or lack of effectiveness.

Finally, a word for the use of LAI clinics: progesterone/LAI clinics for women have been initiated in the past and seem like an excellent service but may not have been reported on. The same applies to LAI-APs clinics, where information is lacking. A potential problem with these is that the patient is potentially not seen by a physician for a long time, and new symptoms as well as side effects may not be noticed and corrected, and this might occur more often with women. Nevertheless, the skills and experience associated with these clinics can be put to best use by incorporating them into maintenance medication clinics, thereby helping to maintain compliance and reduce relapse rates for those LAI medication with or without oral medication.^57^ The most relevant topics discussed in section ‘Sex differences with antipsychotics’ can be found in Summary Table 2.

**Summary Table 2.** Sex differences with LAI-APs.

Sex differences with LAI-APs	
Pharmacokinetics and pharmacodynamics	• Several pharmacokinetic differences exist between men and women treated with LAI-APs, but little is known regarding their pharmacodynamics.
Treatment response and tolerability	• The efficacy and tolerability of APs differs between men and women, and some of these differences are more noticeable depending on the patient’s age and the phase of the illness. • Better responses to most APs have been reported in women, but side effects are more prevalent in women. • Given the relevance of side effects associated with hyperprolactinaemia, prolactin-sparing APs may be preferred for women with SZ. However, whether these APs prevent bone mineral density loss is controversial.
Prescription	• Treatment discontinuation is common in SZ, regardless of sex. However, in the USA and Europe, women with SZ are prescribed LAI-APs less frequently. • Prescribing patterns differ between men and women, which appears to be a result of both physician and patient preferences.
Patients’ preferences	• It is unknown whether attitudes towards LAI-APs differ by sex among individuals with SZ.

LAI-APs, long-acting injectable antipsychotics; SZ, schizophrenia.

### Considerations regarding pregnant, postpartum, breastfeeding and postmenopausal women

#### Pregnancy

APs themselves are not significantly associated with an increased risk of stillbirth, small/large-for-gestational-age births, preterm delivery or spontaneous miscarriage.^12^ However, since clinical trials usually exclude pregnant women, data on the foetal risks of APs are limited and based mainly on case reports. Nevertheless, the potential benefits of APs may outweigh their potential risks of discontinuation during pregnancy.^12,58^

Physiological changes during pregnancy, together with an increase in the blood–brain barrier permeability leads to higher drug bioavailability.^12^ Renal excretion increases during pregnancy, resulting in lower plasma concentrations of APs such as amisulpride, sulpiride and paliperidone.^59^ CYP activity also changes, and this may cause reductions in plasma levels of quetiapine and aripiprazole of more than 50% in the third trimester. Recent studies confirmed that changes in drug blood levels are very common among pregnant women, and decrease progressively over pregnancy^60^; therefore, drug monitoring and blood level assessment should be considered throughout pregnancy especially in the third trimester.^61^

Pregnant women taking APs have been reported to have up to a 20.9% higher risk of developing gestational diabetes, especially when using olanzapine, risperidone, clozapine or high doses of quetiapine (>300 mg/day),^62^ although evidence remains insufficient.^63,64^ Pregnant patients should, thus, be monitored to prevent metabolic complications, with glucose tolerance testing and assessment of cholesterol and triglycerides.^65^

Regarding the use of LAI-APs during pregnancy, there is a tendency for clinicians to either not begin or to even discontinue them, even in women with extremely high risk for relapse.^58^ In fact, women with severe psychiatric illness who are nonadherent during the first trimester are almost twice as likely to relapse as compared to women who are adherent.^66^ Therefore, it has been suggested that the appropriate patient to receive a LAI during pregnancy should not differ significantly from the appropriate patient to receive a LAI when they are not pregnant.^58^ Being treated with a LAI-AP formulation during pregnancy might assure clinical stability and better functionality, greater autonomy, better productivity in activities oriented to self-care during pregnancy, greater quality of life and adequate care for the mother and the newborn after delivery.^67^

LAI-AP formulations are associated with a more constant plasma drug level and may thus reduce foetal exposure to the highly fluctuating plasma drug levels associated with maternal oral AP use.^68^ On the other hand, during pregnancy, the clinician should monitor for the need of a temporarily increased dosage of some APs given pregnancy-associated pharmacokinetic changes,^69^ which may lead to significant differences in plasmatic level, such as with aripiprazole.^70^ This need for dose adjustments throughout pregnancy with the oral formulation of the ongoing LAI-AP may be cumbersome.

LAI-APs with more favourable metabolic profiles should also be selected during pregnancy, given the association of some APs with higher rates of metabolic complications in this period, such as preeclampsia and eclampsia.^71^

Due to ethical reasons, there is a lack of randomized clinical trials examining the safety of LAI-APs during pregnancy and puerperium, and the literature is limited to case reports.^72^ Given that evidence based on case reports is inconclusive, risks should be extrapolated from research on oral APs in pregnancy.^73^

Case reports have been published on the use of palmitate paliperidone monthly LAI during pregnancy,^74,75^ and on the 3-monthly formulation,^76^ as well as on the use of risperidone LAI, the precursor of paliperidone,^77^ with no health or neurodevelopmental problems observed in the infants/newborns.

Therefore, when choosing LAI-APs during pregnancy, the same factors as when treating the nonpregnant patient should be considered, namely: previous efficacy of the oral formulation, ability to be compliant with the needed time for overlap with the oral formulation, potential side effects and cost.^49^ Since many patients with SZ have reduced insight and acceptance of their condition, the role of the family, or other significant ones, is key to involve the patient in the decision to take a LAI-AP, and regarding pregnancy, this discussion should ideally involve the patient’s partner.^58^

#### Postpartum

In the first few weeks after delivery, women with a history of psychosis are at an increased risk of postpartum psychosis due to hormonal, neuro-immune changes and psychosocial factors.^78,79^ It is important to closely monitor clinical symptoms of SZ in this period and it is crucial to continue AP treatment, especially in women taking D2 dopamine agonists, such as bromocriptine, to supress lactation.^80^

LAI-APs with longer dosing intervals should be considered in this phase, since women in general, and those with SZ also, often may have difficulty attending appointments in the postpartum period.^81^

#### Breastfeeding

The benefits of breastfeeding may outweigh the potential risks of AP exposure in the milk, the amount excreted into the breastmilk to the infant is relatively low,^82^ APs are rarely associated with adverse events to the infant/newborn.^67,83–85^ Therefore, women with SZ are encouraged to breastfeed, unless they are taking clozapine.^86^

Regarding LAP-APs formulations, special caution should be taken in cases of women at risk for preterm delivery. Having SZ and the exposure to SGAs itself may be related to a slight increase in the risk of preterm birth and low birth weight^87^; in these cases, the clinician may consider avoiding administering an LAI to women who strictly plan to breastfeed as preterm infants are at risk for elevated plasma medication concentrations due to immature hepatic and renal systems.^88^ Impact on prolactin, a key hormone in the lactation process, should also be considered; aripiprazole a partial agonist of the dopamine receptor often reduces prolactin levels,^89^ and may negatively affect lactation. In contrast, all other LAI-APs are dopamine antagonists and can cause increased prolactin levels. Though increased prolactin levels may not impair lactation, there is the rare possibility of overproduction leading to mastitis, and AP-induced mastitis has been described even in nonpregnant and nonlactating women.^90^

#### Postmenopausal women

Menopause can have a significant impact on women with SZ-spectrum disorders, leading to changes in symptomatology, cognitive function and quality of life.^91^ Oestrogen, via its effect on serotonin, glutamate, noradrenergic and cholinergic pathways, is thought to be relevant to psychosis,^19^ and to enhance AP efficacy.^33^ Whenever oestrogen levels in women decline, psychotic symptoms increase. At menopause, this can happen in two ways: (1) the loss of oestrogen (mainly estradiol) can directly affect central neurotransmission, leading to increase in SZ-related symptoms and (2) the loss of oestrogen can decrease the synthesis of enzymes that metabolize AP drugs.^33^ The much lower oestrogen levels decrease CYP1A2 induction, leading to more rapid conversion of APs that are mainly metabolized by this enzyme (i.e. clozapine and olanzapine).^32^ Therefore, menopausal women become less responsive to APs than they were during their reproductive life,^33^ and their clinical status may worsen.^11,33^ Indeed, more women after the age of 45 need hospitalization, especially in the age group of 65–69.^32^ However, AP dose increase may not be the best strategy, as effectiveness of APs in women over 45 is low for higher dose ranges,^11^ and because it increases the risk of weight gain, cardiovascular and cerebrovascular events.^33^ Changing to an AP that is less affected by oestrogen loss may work better,^92,93^ such as amisulpride, aripiprazole^94^ and possibly cariprazine.

LAI-APs obviate first pass metabolism improving levels of drugs affected by CYP1A2 and CYP3A4 enzymes,^33^ and could, therefore, be an interesting alternative for menopausal women. Prolactin-sparing APs are recommended in menopausal women,^95^ but regarding LAI-APs, this leaves very few options.

Adding hormone replacement or selective oestrogen receptor modulators such as raloxifene, including phytoestrogens (bioidenticals) in the diet, and/or providing psychosocial support, instead of raising the AP dose, may prove effective^33,91^; this could be a good strategy for women on LAI-APs, since dose adjustments with these formulations are not so practical. Summary Table 3 outlines the main considerations regarding the use of LAI-APs in pregnancy, postpartum, breastfeeding and menopause.

**Summary Table 3.** Considerations about specificities of women.

Considerations about specificities of women	
Pregnancy	• Clinical trials of APs usually exclude pregnant and breastfeeding women, so data on the foetal and newborn risks of APs is limited. • Noncompliance with APs during the first trimester is associated with an increased risk of relapse. • LAI-AP formulations may reduce foetal exposure to the highly fluctuating plasma levels associated with oral APs. • Drug monitoring and assessment of AP blood levels may be considered throughout pregnancy, especially in the third trimester. • The choice of LAI-AP during pregnancy should depend on the same factors as when treating nonpregnant patients, including prior efficacy of the oral formulation and potential side effects.
Postpartum and breastfeeding	• Given the risks of relapse during the postpartum period, close monitoring of symptoms and maintenance of AP treatment are warranted. • LAI-AP with longer dosing intervals should be considered during this phase. • Women with SZ receiving APs should not be discouraged from breastfeeding unless they are taking clozapine.
Postmenopausal period	• Menopause is associated with a lower response to APs. • In menopause, clinicians may consider switching to an AP that is less associated with drops in oestrogen levels.
Contraception and parenting	• LAI-APs represent an interesting option for women during contraception and parenting. • Guaranteeing that treatment is being provided by means of a LAI-AP may prove useful in legal settings to assure child custody services of treatment adherence.

LAI-APs, long-acting injectable antipsychotics; SZ, schizophrenia.

### Considerations on contraception and parenting

#### Contraception

One concern with women with SZ is the underuse of contraception, an issue that clinicians frequently fail to address, unless women are married or partnered.^18^ However, women with SZ are sexually active, usually outside of a stable partnered relationship, with a 24.3%–47.5% prevalence of unwanted pregnancy in this population.^96^ Even when under contraception, the hormone levels in oral contraceptives are decreased by antiepileptic drugs,^97^ which sometimes are used as adjunctive treatment in SZ. Moreover, because women with SZ may be unreliable about taking a daily contraceptive pill, other contraceptive methods such as intrauterine devices, depot injections of progesterone and tubal ligation, may be the preferred choice.^98^

LAI-APs could also prove useful in this regard, and in those women on depot injections of progesterone the LAI-AP could even be administered simultaneously, thus reducing the number of appointments for treatment administration. LAI-APs which are dopamine antagonists can cause increased prolactin levels and may prove useful in reinforcing contraception, but not in those cases when the woman and her partner wish to get pregnant. Therefore, all these options should be discussed with the clinician, and whenever possible, in close articulation with the obstetrician.

#### Parenting

Many women with SZ do want to have children, and because the disease begins later in women than in men, many of them are already mothers at symptom onset.^11^ While some have partners and families who help with the task of parenting, others are single mothers.^99^ Symptoms of SZ may disrupt parenting behaviours and tend to become more severe in the postpartum period.^78,79^ Mothers with SZ usually face economic hardships, housing difficulties, a lack of family and informal social support networks and access to meaningful legal representation, and approximately 50% end up losing custody of their children.^100^ This is partly because there is a general belief that a diagnosis of SZ is incompatible with adequate child care and that SZ mothers are more prone than others to neglect or abuse their children.^14^

Because of raising small children at home, mothers with SZ may neglect their health, miss doses of oral treatment and fail medical appointments. Since they may have more difficulty to leave the house, available at home services must be provided, including medical and nursing care^14^; this could be achieved by the use of LAI-APs, which simplify the treatment regimen and allows for guaranteeing that treatment is being provided. This could also prove useful in legal settings to assure child custody services of treatment adherence. Summary Table 3 compiles the main considerations on contraception and parenting.

#### Forensic aspects

One reason why LAI-APs are less used in women is because women are usually perceived as less aggressive than men. Aggressive behaviour represents a challenge in the treatment of patients with SZ, being often associated with illness severity, poor insight, substance misuse or comorbidity with personality disorders. As psychotic relapses and consequent risk of aggressive behaviour are often associated with poor compliance, LAI-APs could play a promising role in the management of aggressive behaviour of subjects with SZ.

Although the sex ratio of criminal acts between women and men has been described as high as 1:10,^101^ violent and nonviolent patients have also been found to be comparable in terms of sex.^102^

Aside from clozapine, LAI-APs appear to be superior to oral APs for preventing violence,^103^ and have been shown to significantly reduce the severity of hostility, aggressivity, number of violent incidents and criminal offences.^104^

Therefore, data encourage the use of LAI-APs in forensic psychiatry, and being a woman should not hinder their use.

## Final considerations

Men and women are different, and sex differences in SZ are nowadays scientifically established.^14,17^ Efficacy and tolerability of APs is significantly different between men and women with SZ,^12^ and some of these differences are more noticeable depending on the age and stage of the disorder,^20^ with optimal AP treatment for women being highly dependent on the different life phases.

Taking sex aspects into account when treating SZ patients is now recommended,^105^ and the European Medicines Agency decided to foster the development for separate guidelines for women in clinical trials,^106^ which remains an under researched area, especially concerning LAI-APs.^16^

Treatment guidelines have been very conservative on the use of LAI-APs,^6,7^ especially regarding their early implementation,^76^ and so, despite their benefits, LAI-APs remain underutilized in patients with SZ, most notably in women.^9–12^ The dearth of information regarding their use in (a potential) pregnancy might explain clinician fears. Little is known about the best strategies to adjust dosages throughout the woman’s lifecycle, and even within the ovulatory cycle. Nevertheless, the advantages seem to surpass the possible disadvantages, and therefore women should maintain AP treatment during their lifecycle with no interruptions.

Women prescription rates of LAI-APs are lower than in men,^9–11^ although they may benefit as much or even more from these formulations. Therefore, future trials on LAI-APs should compare the effectiveness not only between men and women, but also between pre- and postmenopausal women,^33^ stratifying by hormonal status when analyzing the results.^107^ This information should be used in a shared-decision process, involving the patients and their families/partners, to facilitate LAI-APs’ use in a manner that is constructive to the clinician–patient relationship and successful treatment.^49^ Until such information is attainable for tailoring treatment decisions, the patient’s sex is valuable immediately available information on which to personalize treatments,^108^ and sex-specific drug selection and dose determination has the potential to reduce the overall adverse effects of treatments in clinical practice,^108^ namely with LAI-APs. (Summary Table 4).

**Summary Table 4.** Recommendations on the use of LAI-APs in women with schizophrenia.

Recommendations
• Personalized treatment of SZ considering sex differences, represents a priority in the management of this illness. • Pregnancy should not discourage clinicians from prescribing LAI-APs, and avoiding or discontinuing LAI-APs during pregnancy in women at high risk of relapse is not recommended. • Consider avoiding LAI-APs in women at risk of preterm birth and those who plan to strictly breastfeed. • Prolactin-sparing APs are recommended in menopausal women, but this allows very few options regarding LAI-APs. • Future clinical trials should compare the efficacy of LAI-APs between men and women with SZ, as well as between pre- and postmenopausal women, stratifying outcomes by sex and hormonal status, respectively. • Sex differences should be considered in clinical practice guidelines regarding treatment recommendations on LAI-AP.

LAI-APs, long-acting injectable antipsychotics; SZ, schizophrenia.

## Declarations
